# A new approach to predict soil temperature under vegetated surfaces

**DOI:** 10.1007/s40808-015-0041-2

**Published:** 2015-10-29

**Authors:** Klaus Dolschak, Karl Gartner, Torsten W. Berger

**Affiliations:** Department of Forest- and Soil Sciences, Institute of Forest Ecology, University of Natural Resources and Live Sciences (BOKU), Peter Jordan-Straße 82, 1190 Vienna, Austria; Department of Forest Ecology and Soil, Federal Research and Training Centre for Forests, Natural Hazards and Landscape, Seckendorff-Gudent-Weg 8, 1131 Vienna, Austria

**Keywords:** Empirical model, Dynamical model, Newton’s law of cooling, Forest soil temperature, Freeze/thaw transition, Simulated annealing

## Abstract

In this article, the setup and the application of an empirical model, based on Newton’s law of cooling, capable to predict daily mean soil temperature (*T*_soil_) under vegetated surfaces, is described. The only input variable, necessary to run the model, is a time series of daily mean air temperature. The simulator employs 9 empirical parameters, which were estimated by inverse modeling. The model, which primarily addresses forested sites, incorporates the effect of snow cover and soil freezing on soil temperature. The model was applied to several temperate forest sites, managing the split between Central Europe (Austria) and the United States (Harvard Forest, Massachusetts; Hubbard Brook, New Hampshire), aiming to cover a broad range of site characteristics. Investigated stands differ fundamentally in stand composition, elevation, exposition, annual mean temperature, precipitation regime, as well as in the duration of winter snow cover. At last, to explore the limits of the formulation, the simulator was applied to non-forest sites (Illinois), where soil temperature was recorded under short cut grass. The model was parameterized, specifically to site and measurement depth. After calibration of the model, an evaluation was performed, using ~50 % of the available data. In each case, the simulator was capable to deliver a feasible prediction of soil temperature in the validation time interval. To evaluate the practical suitability of the simulator, the minimum amount of soil temperature point measurements, necessary to yield expedient model performance was determined. In the investigated case 13–20 point observations, uniformly distributed within an 11-year timeframe, have been proven sufficient to yield sound model performance (root mean square error <0.9 °C, Nash–Sutcliffe efficiency >0.97). This makes the model suitable for the application on sites, where the information on soil temperature is discontinuous or scarce.

## Introduction

Various biotic, as well as abiotic processes in the soil are temperature dependent (Rankinen et al. 2004). Usually, these dependencies are assumed to have a non-linear nature (Bond-Lamberty et al. 2005; Davidson et al. 2006; Macdonald et al. 1995; Wagle and Kakani 2014), meaning that the response of the process to changes of temperature, strongly depends on the temperature range it is occurring in. Especially for high temperatures, small changes in temperature might yield big changes in the processes response. For the assessment of temperature dependent soil processes, it is therefore crucial to have expedient knowledge about spatial, as well as temporal fluctuations of soil temperature (Bond-Lamberty et al. 2005). The most reliable source of information would be the permanent monitoring of subsurface ground temperature. But in practice it is often hard to measure continuously. Usually, the modeler has to deal with fragmentary timelines of soil temperature, scarce point observations or even no records of *T*_soil_ at all (Lei et al. 2011). To fill these gaps or to extend the timeline beyond the measurement timeframe, the researcher has to consider the application of a soil temperature model.

The approaches to predict subsurface ground temperature can be coarsely divided in 2 categories; (1) process based models, and (2) empirical models (Kang et al. 2000). Process based approaches to predict soil temperature generally use meteorological input variables (primarily temperature and solar radiation) to calculate energy balance of the soil surface, and heat transport in the soil, by solving the heat equation (Paul et al. 2004). The applicability of these models is often limited by their high complexity, high demand of input data, and specific model parameters, which are often not available for the investigated site (Lei et al. 2011; Svensson et al. 2008). Empirical models, presented in the work of Brown et al. (2000), Kang et al. (2000), or Paul et al. (2004), rely on the statistical relationship between meteorological parameters and soil temperature. More recently, there have been successful attempts to predict *T*_soil_ using combinations of artificial neural networks and fuzzy logic (Bilgili et al. 2013; Kim and Singh 2014; Kisi et al. 2015; Talaee 2014).

Soil thermal regimes are controlled by various environmental drivers. The most important meteorological factors are air temperature and radiation, laying the base for heat exchange at the soil surface (Hu and Feng 2003). In the latter, forested sites differ substantially from other types of land-cover: The radiation driven heat exchange between soil surface and atmosphere, is limited due to the shielding effect of the canopy (Paul et al. 2004). Therefore, forested sites show strongly dampened *T*_soil_ fluctuations, compared to sites with sparse vegetation or bare soil (Balisky and Burton 1993). Only a few models exist, which explicitly address the soil thermal conditions of forested ecosystems.

Zheng et al. (1993) set up a dynamical *T*_soil_ model based on Newton’s law of cooling, assuming the change of *T*_soil_ proportional to the temperature difference between air and soil. The fact, that the vegetation cover limits radiation driven heat flux, is taken into account by utilizing a heat transfer coefficient, which depends on the stands leaf area. They assume, that the canopy’s damping effect is more pronounced for incoming radiation, than for emission from the ground. This is incorporated, by applying different heat transfer coefficients, whether the soil is warming or cooling. The damping term, dependent on LAI, only comes into effect for soil warming conditions. Based on this work, Kang et al. (2000) set up a spatially resolved *T*_soil_ model. To describe the soil thermal regimes of South Korean forest sites, they extended the latter approach by introducing a more ‘mechanistic’ element, based on Fourier’s law of heat transport. Besides the spatial and temporal variability of the leaf area, this approach also accounts for the effect of the stands litter layer on soil heat flux. The authors assumed, that *T*_soil_ does not fall below freezing for most Korean forest sites. As well as in the latter approach, *T*_soil_ estimates below 0 °C were replaced with 0 °C.

Brown et al. (2000), predicted daily mean *T*_soil_ of 4 different Northern Hardwood stands, utilizing a statistical relationship between *T*_soil_ and the average air temperature of the previous day. As a correction term, accounting for the phase shift or ‘lagging behind’ of the annual course of *T*_soil_ compared to air temperature, they introduced a cosine function of the Julian day. Despite the simple model structure, the predictions of *T*_soil_ were quite precise (disregarding the cold season).

To predict daily *T*_soil_ of various Australian forest sites, Paul et al. (2004) used daily average air temperature and stand parameters like leaf area, understory growth, and litter mass. They assumed *T*_soil_ oscillating around an annual mean soil temperature, which is calculated from annual mean air temperature, modified with a correction factor, derived from information about the stands’ vegetation cover and litter layer. The resulting temperature wave is then offset by a term describing daily fluctuations of *T*_soil_, which again, is derived from air temperature. The model specifically addresses the thermal conditions of the topsoil. Therefore, phase shift and attenuation of the temperature oscillation, which become relevant with increasing soil depth, were not considered.

Bond-Lamberty et al. (2005) examined the spatiotemporal dynamics of soil thermal regimes during stand development of a disturbed boreal forest. To accompany this investigation and for laying the base to simulate forest dynamics, they implemented an empirical *T*_soil_ model. Accounting for the influence of recent past air temperature conditions on present *T*_soil_, they calculate running averages of the daily mean air temperature. *T*_soil_ is then calculated as a linear function of multiple running averages, centered to different days in the past. The authors report difficulties to predict *T*_soil_ close to the freeze/thaw transition.

To evaluate the suitability of *T*_soil_ as a predictor for the treeline position in the Swiss Alps, Gehrig-Fasel et al. (2008) presented an approach, which strongly differs from others described in this section. To satisfy the statistical requirements for regression modeling, the data was first detrended and then transformed for first differences. After performing the regression analysis, the data was transformed back. Considering that daily mean air temperature was the only input parameter, the model showed high performance in the validation timeframe. Assuming only an insignificant influence of winter soil temperatures on the treeline position (Körner and Paulsen 2004), the validation could be limited to the warm season.

Most approaches presented here disregard *T*_soil_ dynamics of the cold season. The decoupling of the soil from the atmosphere by a fluctuating snowpack (Betts et al. 2001), the heat transformation processes at the phase change from liquid to frozen (Beltrami 2001; Viterbo et al. 1999), or changes in heat capacity and conductivity seem difficult to be captured in the framework of an empirical approach. In cases where winter *T*_soil_ is assumed to reach or fall below 0 °C, process based approaches, presented by e.g. Rankinen et al. (2004), should be preferred. But, even though this model could be described ‘simple’ from a mechanistic point of view, solely the empirical snow accumulation/melt module, upstream to the *T*_soil_ model, requires the assignment of 11 free parameters. An alternative might be the semi-empirical model presented by Katterer and Andren (2009). Making the approach suitable for colder temperature conditions, the formulation presented by Kang et al. (2000) was modified. They interposed a surface temperature term, which acts as link between air and soil temperature. In this term the influence of air temperatures below 0 °C is attenuated by a constant factor. This way, they account for the low thermal conductivity of snow.

The objective of this article is the presentation of a model to predict soil temperature of forest stands, which aims to perform like a ‘well-tuned’ mechanistic simulator, using the straightforwardness of an empiric formulation. The model enables the transformation of fragmentary records of forest soil temperature, into a complete time series of *T*_soil_, using average daily air temperature as only input. In this specific case, the created time series is laying the base for the modeling of temperature dependent, biogeochemical soil processes. Due to the fact that many biotic soil processes are sensitive to winter conditions (Campbell et al. 2005), emphasis is laid on an expedient representation of the temperature dynamics of the cold season.

Running the simulation requires the adjustment of nine empirical parameters, which are not defined in a strict physical sense. This is making it hard to deduce parameter values directly from site information. For a proper site specific parameterization, at least some snapshot measurements of *T*_soil_ are recommended. Therefore, this model primarily aims to sites were *T*_soil_ data is available, but the time series are inconsistent, or have to be extended beyond the timeframe of measurement.

## Materials and methods

### Model description

The model describes *T*_soil_ as a function of daily mean air temperature (*T*_air*,t*_). It employs a daily time step. The formulation is based on Newton’s law of cooling (Bergman et al. 2011), which is applied 2 times consecutively.

Utilizing a relatively small heat transfer coefficient (*λ*_shift_), the first application of Newton’s law provides a phase shifted temperature time series (*T*_shift*,t*_) which lacks the high frequency fluctuations of *T*_air*,t*_.

1 $$T_{{{\text{shift,}}t}} = T_{{{\text{air,}}t}} + \left( {T_{{{\text{shift,}}t - 1}} - T_{{{\text{air}},t}} } \right)\exp \left( { - \lambda_{\text{shift}} } \right)$$

A fictive environmental temperature (*T*_env*,t*_) is postulated as the weighted mean of the elements *T*_air*,t*_, *T*_shift*,t*_, and a constant correction temperature (*T*_corr_). *pc*_air_, *pc*_shift_, and *pc*_corr_ are partitioning coefficients, which define the relative weight of the specific element.

2 $$T_{{{\text{env}},t}} = T_{{{\text{air}},t}} pc_{\text{air}} + T_{{{\text{shift}},t}} pc_{\text{shift}} + T_{\text{corr}} pc_{\text{corr}}$$

The partitioning coefficients sum up to 1, so 2 have to be defined as model parameters, one can be deduced.

3 $$pc_{\text{corr}} = 1 - \left( {pc_{\text{air}} + pc_{\text{shift}} } \right)$$

Δ*T* states the difference of the soil temperature to *T*_env*,t*_.

4 $$\Delta T = T_{{{\text{env,}}t}} - T_{{{\text{soil}},t - 1}}$$

**Fig. 1:**
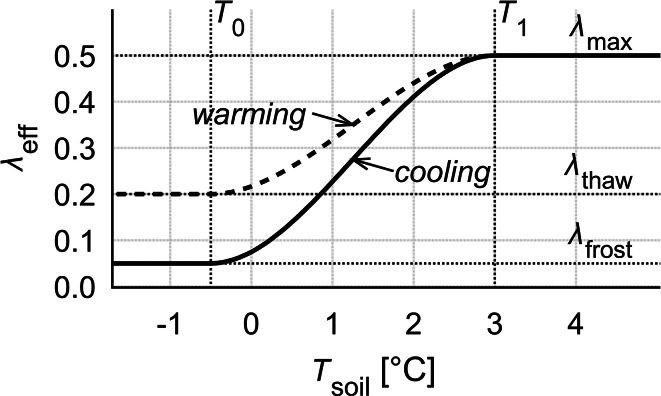
Polynomial transition of the heat compensation coefficient (*λ*_eff_), between 2 threshold soil temperatures (*T*_0_, *T*_1_), close to soil freezing. The reduction of the coefficient pays respect to the energy release/demand of phase changes, from liquid to solid and vice versa. High model performance was achieved, using different minimal compensation coefficients for soil cooling (*λ*_frost_) (*solid line*) and warming (*λ*_thaw_) (*dashed line*) respectively

Taking into account the insulating effect of the snow cover and the heat release/consumption due to the phase change of soil water from liquid to solid and vice versa (Beltrami 2001), a variable heat transfer coefficient (*λ*_eff_) is implemented (Fig. 1). *λ*_max_ represents the transfer coefficient above the upper threshold temperature (*T*_1_). Below *T*_1_*λ*_eff_ gets reduced, reaching the minimum (*λ*_min_) at the lower threshold (*T*_0_), where different *λ*_min_ are applied for soil warming and cooling.

5 $$\lambda_{\hbox{min} } = \left\{ {\begin{array}{*{20}r} \hfill {\lambda_{\text{thaw}} ,} & \hfill {\Delta T > 0} \\ \hfill {\lambda_{\text{frost}} ,} & \hfill {\Delta T \le 0} \\ \end{array} } \right.$$

The transition of the transfer coefficient in between*T*_1_ and*T*_0_ is described, using a third order polynomial.

6 $$\lambda_{\text{eff}} = \left\{ {\lambda_{ \hbox{min} } + \left( {\lambda_{ \hbox{max} } - \lambda_{ \hbox{min} } } \right)\begin{array}{ll} \lambda_{ \hbox{min} } , & T_{{{\text{soil}},t - 1}} \le T_{0} \\ \left( {3x^{2} - 2x^{3} } \right), & T_{0} < T_{{{\text{soil}},t - 1}} < T_{1} \\ \lambda_{ \hbox{max} } , & T_{{{\text{soil}},t - 1}} \ge T_{1} \\ \end{array} } \right.$$

*T*_soil*,t*−1_ has to be transformed into an auxiliary variable inside the interval 0–1.

7 $$x = \frac{{T_{{{\text{soil}},t - 1}} - T_{0} }}{{T_{1} - T_{0} }}$$

At last, Newton’s law is applied the 2nd time. The actual daily mean soil temperature calculates as:

8 $$T_{{{\text{soil}},t}} = T_{{{\text{env}},t}} - \Delta T { \exp }( - \lambda_{\text{eff}} )$$

### Study sites/input data

#### Austria

In the framework of the International Co-operative Programme on Assessment and Monitoring of Air Pollution Effects on Forests (ICP Forests), the Austrian Research Centre for Forests operates several, intensively monitored, forest sites (Level II) (Neumann et al. 2001). In addition to various other environmental parameters, meteorological conditions are monitored continuously. Soil temperature records exist for soil depths, ranging from 5 to 60 cm.

The model was originally set up on data from the Level II Plot Klausen-Leopoldsdorf, which is located in the Vienna Woods (48°07′16″N, 16°02′52″E), at an elevation of 510 m a. s. l. The research site is a pure beech (*Fagus sylvatica* L.) stand, which was planted in the late thirties of the last century. The location is facing NE with an inclination of 20 %. The actual forest vegetation coincides with the potential natural one, and can be classified as Hordylemo-Fagetum (Mucina et al. 1993).

**Fig. 2:**
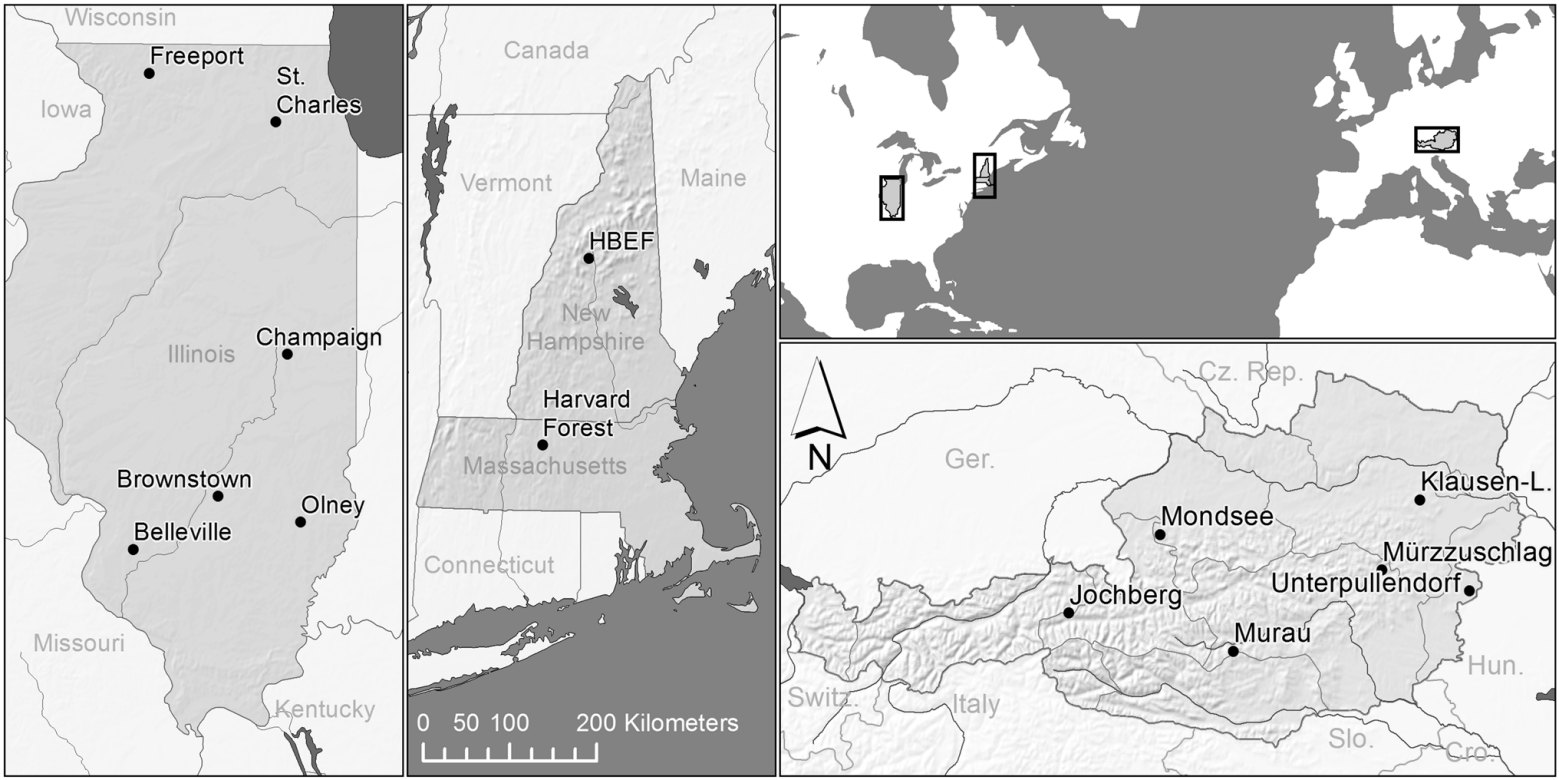
Location of study sites in the United States and in Austria. The sites used for parameterization of the forest soil temperature simulator cover a broad range of characteristics. For a brief site description see Table 1

Subsequently data from 5 other Level II forest stands were accessed (Fig. 2, Table 1). The selection aims to cover a broad range of site characteristics. Investigated sites show a strong altitudinal and climatic gradient. The elevation of the investigated stands ranges from 290 (Unterpullendorf) to 1540 m a.s.l. (Murau), leading to annual mean temperatures from 9.6 to 5 °C, respectively. Austria lies in the transition zone between oceanic and continental climate. Progressing from west to east, investigated locations therefore experience a strong decline in annual precipitation sums, ranging from 1521 mm for mountainous stands in the north-west, affected by orographic precipitation (Mondsee), to 630 mm in the continentally influenced east of the country (Unterpullendorf).

To fill gaps in the record of average daily air temperature, data were accessed, provided by the European Climate Assessment (ECA&D) (Tank et al. 2002). Missing values were replaced, using linear regression with available neighboring stations.

#### East Coast of the United States

Intending to test the models over regional validity, the continent was switched. Data were accessed from 2 intensive long-term ecological research areas in New England; (1) the Hubbard Brook Experimental Forest (HBEF), and (2) Harvard Forest (Fig. 2, Table 1).

The HBEF is located in the White Mountain National Forest in north-central New Hampshire (43°56′N, 71°42′W). The elevation of the investigated watershed ranges from 250 m to 1000 m. The forest type can be classified as Northern Hardwood, dominated by Sugar maple (*Acer saccharum* Marsh). The climate is cool, continental, and humid, with mean annual precipitation sums around 1400 mm (Bailey et al. 2003). Approximately one-third of the precipitation is falling as snow, leading to a snowpack, typically lasting from December to April. Soils can be classified as well-drained Spodosols (WRB: Podzol), developed on glacial till (Campbell et al. 2010).

Daily *T*_soil_ (depth: 5 cm) data, recorded within the framework of the project ‘Snow Depth & Soil Freezing as a Regulator of Microbial Processes’ (Duran et al. 2014), were obtained. Data of 3 intensive high elevation plots (mean elevation: 560 m, exposition: North) and 3 intensive low elevation plots (mean elevation: 430 m, exposition: South) were used. For each altitude class, one mean time-series of *T*_soil_ was calculated.

Because of their proximity to the investigated stands, records of air temperature (Bailey et al. 2003) from meteorological station 23 and 1, for high and low elevation plots respectively were obtained. Missing data were replaced, using offset temperatures of highly correlated neighboring stations. To fill remaining gaps in the air temperature record, the GHCN-Daily dataset was accessed, provided by the NOAA (Menne et al. 2012a, b), utilizing data from the station Wentworth, New Hampshire (43°52′22″N, 71°54′31″W).

The Harvard Forest Research Station is located in Central Massachusetts (42°32′N, 72°11′W). The climate is cool, temperate, and humid. Precipitation is distributed evenly through the year, with annual sums in the range of 1080 mm. The annual mean temperature is 8.5 °C (Berbeco et al. 2012). The elevation of the investigated locations is approximately 350 m a. s. l. Soils can be classified as Typic Dystrudepts (WRB: Dystric Cambisol). After a severe disturbance in the beginning of the last century, the forest regrew naturally, resulting in an even aged stand of mixed hardwood species, with Red oak (*Quercus rubra* L.) dominating (Butler et al. 2012).

Within the forest site, the simulator was applied to 2 sub-sites: (1) Barre Woods (Melillo et al. 2003), and (2) Prospect Hill (Melillo et al. 1999). Both locations were set up to study the effect of soil warming on carbon and nitrogen turnover, by artificially heating the ground (Berbeco et al. 2012; Melillo et al. 2002). The model was adjusted to the topsoil (depth 5 cm) of the undisturbed control plots, whereat on the Prospect Hill site data from 6 control plots were combined, calculating a mean time-series of *T*_soil_. Daily air temperature was obtained from the EMS tower (Munger and Wofsy 1999), where the record 7.6 m above ground was selected. Data gaps were closed, using offset temperature measurements at other heights, or data from the Fisher meteorological station (Boose 2001). If no other source was available, the GHCN-Daily dataset was again accessed, applying offset air temperature data from the Municipal Airport station at Orange, Massachusetts (42°33′46″N, 72°16′59″W).

#### Non-forested sites in Illinois

At last, to explore the limits of the formulation, the model was applied to 6 sites which lack the shielding properties of a dense forest canopy. Therefore, data were obtained from the Illinois Climate Network, (ICN), which operates several open field meteorological stations in Illinois  (Fig. 2, Table 1). Air temperature was measured 2 m above ground. Gaps in the air temperature record were closed, using offset temperature measurements of, highly correlated, and neighboring stations. Soil temperature was recorded in 10 cm and 20 cm depth (Hollinger et al. 1994) under sod covered ground. Soil texture was assessed as silt loam, throughout all studied locations. The elevation of the investigated sites ranges from 133 to 265 m a. s. l.

Illinois’ climate is typically continental with cold winters and warm summers. Moving from north to south, mean annual air temperatures increase from 8.9 to 14.5 °C. Also annual precipitation sums reveal a strong north–south gradient, ranging from 810 to 1220 mm. Stations in the north-west of the state are climatically influenced by Lake Michigan, which is attenuating temperature extremes and enhancing winter precipitation (lake effect snow) (Changnon et al. 2008).

### Model application

#### Parameterization

The model was applied to each site and depth specifically. Emphasis was laid on its application on longest possible records of *T*_soil_, to cover the broadest possible range of different environmental states, which might have a potential influence on soil thermal regimes. On the other hand it seems obvious, that due to changes in leaf area, undergrowth, litter layer, water consumption, etc., forest *T*_soil_ regimes undergo a certain shift during stand development (compare Kang et al. 2000). In cases where, for reasons unknown, an obvious change in the soil thermal regime was observed, the time frame of the investigation was manually narrowed down. Both *T*_soil*,t*_ and *T*_shift*,t*_ were initialized at 8 °C. The simulator ran a 150 day spin-up prior to the analysis time frame. For model parameterization a simulated annealing algorithm (Kirkpatrick et al. 1983) was applied, selecting an exponential cooling schedule. Optimization/evaluation criterion was in every case the Nash–Sutcliffe model efficiency (NSE) (Nash and Sutcliffe 1970).

9 $$\varvec{NSE} = 1 - \frac{{\mathop \sum \nolimits_{{\varvec{i} = 1}}^{\varvec{n}} \left( {\varvec{T}_{{{\mathbf{soil}},\varvec{obs},\varvec{i}}} - \varvec{T}_{{{\mathbf{soil}},\varvec{sim},\varvec{i}}} } \right)^{2} }}{{\mathop \sum \nolimits_{{\varvec{i} = 1}}^{\varvec{n}} \left( {\varvec{T}_{{{\mathbf{soil}},\varvec{obs},\varvec{i}}} - \overline{{\varvec{T}_{{{\mathbf{soil}},\varvec{obs}}} }} } \right)^{2} }}$$

Enabling a balanced split, the calibration was conducted on data from odd years, data from even years served in the evaluation. Making the simulation result comparable to other works, other performance indices like Root Mean Squared Error (RMSE), mean absolute error (MAE) and mean bias error (MBE) were calculated.

10 $$\varvec{RMSE} = \left[ {\varvec{n}^{ - 1} \mathop \sum \limits_{{\varvec{i} = 1}}^{\varvec{n}} \left( {\varvec{T}_{{{\mathbf{soil}},\varvec{obs},\varvec{i}}} - \varvec{T}_{{{\mathbf{soil}},\varvec{sim},\varvec{i}}} } \right)^{2} } \right]^{{{\raise0.7ex\hbox{$1$} \!\mathord{\left/ {\vphantom {1 2}}\right.\kern-0pt} \!\lower0.7ex\hbox{$2$}}}}$$

11 $$\varvec{MAE} = \varvec{n}^{ - 1} \mathop \sum \limits_{{\varvec{i} = 1}}^{\varvec{n}} \left| {\varvec{T}_{{{\mathbf{soil}},\varvec{obs},\varvec{i}}} - \varvec{T}_{{{\mathbf{soil}},\varvec{sim},\varvec{i}}} } \right|$$

12 $$\varvec{MBE} = \varvec{n}^{ - 1} \mathop \sum \limits_{{\varvec{i} = 1}}^{\varvec{n}} \varvec{T}_{{{\mathbf{soil}},\varvec{obs},\varvec{i}}} - \varvec{T}_{{{\mathbf{soil}},\varvec{sim},\varvec{i}}}$$

#### Parameterization on limited input data

To test the simulators practical suitability to cope with limited input data, the *T*_soil_ record of Klausen Leopoldsdorf (15 cm depth) was used, ranging from November 2001 to June 2013 (~11 years, 4053 valid observations). The dataset was split into *n* sectors of approximately equal size. The parameterization (simulated annealing) was performed, drawing only one random observation per sector. The remaining observations served in the evaluation. This step was repeated 12 times per *n*, each time with different random observations, to generate a distributed result. After 12 iterations, *n* was incremented, starting with *n* = 4, gradually progressing to *n* = 2000. This way, the minimum number of point observations was determined, necessary to yield satisfactory model performance.

**Table 1:** Investigated locations cover a broad range of site characteristics and distinct climatic and altitudinal gradients

		Elevation (m a. s. l.)	Exp.	Slope (°)	MAT (°C)	MAP (mm)	Dominant species	Soil type
Level II	Jochberg	1050	NE	4	5.7	1358	*Picea abies*	Dystric Cambisol
	Mondsee	860	SE	14	~5.7	1521	*Picea abies*	Eutric Cambisol
	Murau	1540	N	33	5.0	918	*Picea abies*	Dystric Cambisol
	Mürzzuschlag	715	S	10	6.0	933	*Picea abies*	Eutric Cambisol
	Klausen-Leopoldsdorf	510	NE	11	8.2	804	*Fagus sylvatica*	Stagnic Cambisol
	Unterpullendorf	290	–	0	9.6	630	*Quercus petraea/cerris*	Planosol
HBEF	High Elevation Plots	560	N	~13	5.0	1400	*Betula alleghaniensis*	Podzol
	Low Elevation Plots	430	S	~11	6.1	1400	*Acer saccharum*	Podzol
Harvard Forest	Prospect Hill	365	–	0	8.5	1080	*Quercus rubra*	Dystric Cambisol
	Barre Woods	305	–	0	8.5	1080	*Quercus rubra/velutina*	Dystric Cambisol
ICN	Freeport	265	–	0	~9.1	~860	*Sod covered ground*	
	St. Charles	226	–	0	~9.3	~780	*Sod covered ground*	
	Champaign	219	–	0	~11.3	~1020	*Sod covered ground*	
	Belleville	133	–	0	~12.7	~960	*Sod covered ground*	
	Brownstown	177	–	0	~12.3	~960	*Sod covered ground*	
	Olney	134	–	0	~12.5	~1010	*Sod covered ground*	

*MAT* mean annual temperature, *MAP* mean annual precipitation sum

**Table 2:** Parameterization result for 36 sites and depths

	Parameter									
	z (cm)	*λ*_max_	*λ*_aux_	*λ*_frost_	*λ*_thaw_	*T*_0_ (°C)	*T*_1_ (°C)	*T*_corr_ (°C)	*pc*_corr_	*pc*_air_
Jochberg	15	0.4059	0.0365	0.0041	0.0568	1.3	3.6	2.7	0.142	0.505
	30	0.2781	0.0327	0.0044	0.0806	1.5	5.6	3.5	0.181	0.440
	60	0.1349	0.0273	0.0008	0.0942	1.0	9.2	3.2	0.216	0.380
Mondsee	15	0.4653	0.0708	0.0026	0.0700	0.9	7.6	11.2	0.124	0.438
	30	0.3090	0.0541	0.0056	0.0616	1.2	8.3	9.3	0.234	0.306
	60	0.3672	0.0419	0.0084	0.2010	1.7	12.0	8.1	0.334	0.078
Murau	15	0.3686	0.0447	0.0000	0.0285	0.6	7.0	3.4	0.257	0.285
	30	0.2934	0.0424	0.0005	0.0296	0.8	7.0	3.8	0.298	0.247
	60	0.2514	0.0379	0.0037	0.0498	1.5	6.7	3.8	0.350	0.158
Mürzzuschlag	15	0.2494	0.0208	0.0028	0.0140	0.3	2.7	7.4	0.188	0.480
	30	0.1687	0.0184	0.0130	0.0244	0.9	2.7	7.4	0.213	0.451
	60	0.1119	0.0177	0.0031	0.0321	−0.3	6.4	7.1	0.274	0.353
Klausen-Leopoldsdorf	05	0.5092	0.0261	0.0131	0.1840	1.5	5.4	7.5	0.129	0.538
	10	0.3949	0.0244	0.0130	0.1374	1.6	6.2	7.9	0.151	0.500
	15	0.3006	0.0229	0.0123	0.1287	1.5	7.6	7.9	0.168	0.471
	30	0.2104	0.0214	0.0147	0.0947	2.0	8.3	8.2	0.201	0.432
	60	0.1138	0.0204	0.0021	0.0590	0.9	9.3	8.3	0.278	0.349
Unterpullendorf	15	0.4752	0.0383	0.0360	0.0541	−2.3	12.5	13.6	0.137	0.528
	30	0.2824	0.0313	0.0177	0.0191	−3.2	12.3	12.9	0.172	0.460
	60	0.1443	0.0254	0.0091	0.0101	−2.6	9.3	12.0	0.216	0.366
HBEF, intensive high	05	0.6399	0.0418	0.0024	0.0133	1.1	5.3	10.9	0.300	0.411
HBEF, intensive low	05	0.5584	0.0355	0.0001	0.0075	0.9	3.1	11.4	0.286	0.515
Harvard Forest, Prospect Hill	05	0.8723	0.0447	0.0000	0.0116	−0.4	9.2	14.2	0.160	0.516
Harvard Forest, Barre Woods	05	0.7238	0.0467	0.0000	0.0357	0.4	7.0	16.3	0.153	0.502
Freeport	10	0.7111	0.0974	0.0009	0.1094	−0.8	7.3	118.1	0.012	0.495
	20	0.5157	0.0808	0.0014	0.1598	−0.4	10.2	94.5	0.016	0.437
St. Charles	10	0.7541	0.0915	0.0045	0.1397	−0.5	7.1	182.4	0.007	0.573
	20	0.5323	0.0771	0.0035	0.2367	−0.7	10.7	80.4	0.012	0.540
Champaign	10	0.8515	0.1215	0.0000	0.3250	0.2	6.0	264.2	0.008	0.487
	20	0.5223	0.0822	0.0000	0.3130	00.5	8.8	95.2	0.022	0.512
Belleville	10	0.6570	0.0722	0.0072	0.6131	−0.1	11.0	83.8	0.014	0.468
	20	0.4561	0.0598	0.0044	0.4558	−0.1	11.3	68.9	0.017	0.481
Brownstown	10	0.6627	0.0916	0.0003	0.4320	−0.4	13.3	32.6	0.029	0.508
	20	0.4212	0.0754	0.0036	0.2782	−0.3	13.2	22.6	0.046	0.528
Olney	10	0.8012	0.1035	0.0010	0.4226	−0.7	7.7	343.5	0.003	0.544
	20	0.5477	0.0909	0.0015	0.4373	−0.4	10.0	431.5	0.003	0.492

Optimization was performed, using a simulated annealing algorithm. Performance criterion was the Nash–Sutcliffe Efficiency (NSE)

## Results and discussion

**Fig. 3:**
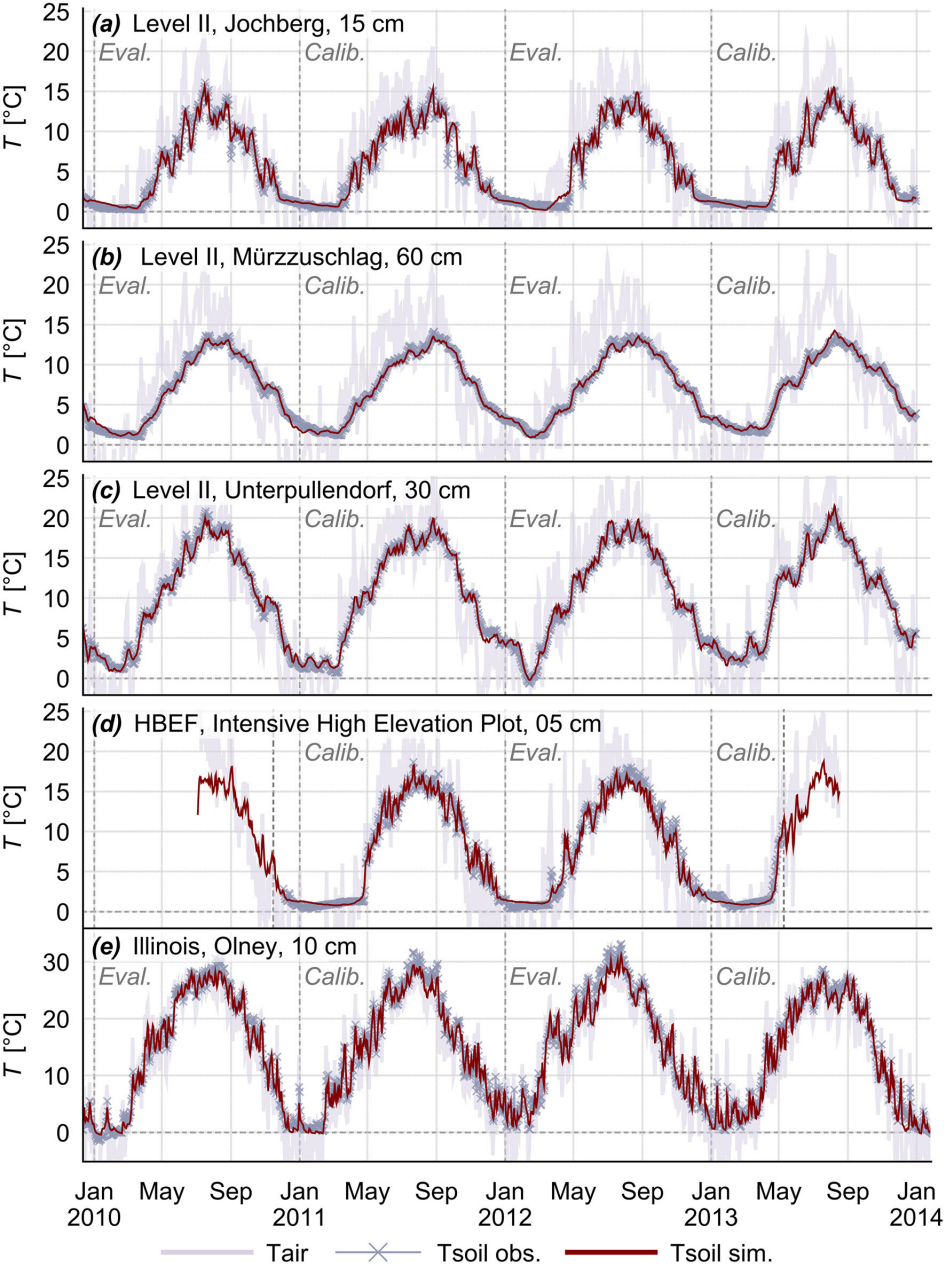
Four years of observed air and soil temperature, overlaid with simulated *T*_soil_. Calibration was performed on odd years, performance evaluation on even ones. Plot (**a**) and (**d**) clearly show the effect of snow cover on winter soil thermal regimes. In both cases the trend was successfully captured by the simulator. Also increasing phase shift and attenuation of the soil temperature wave with increasing soil depth (**b**) were captured. Stronger fluctuations of *T*_soil_ under open-field conditions (**e**), where the heat exchange might be dominated by radiation fluxes, did also not limit the simulators capability

**Fig. 4:**
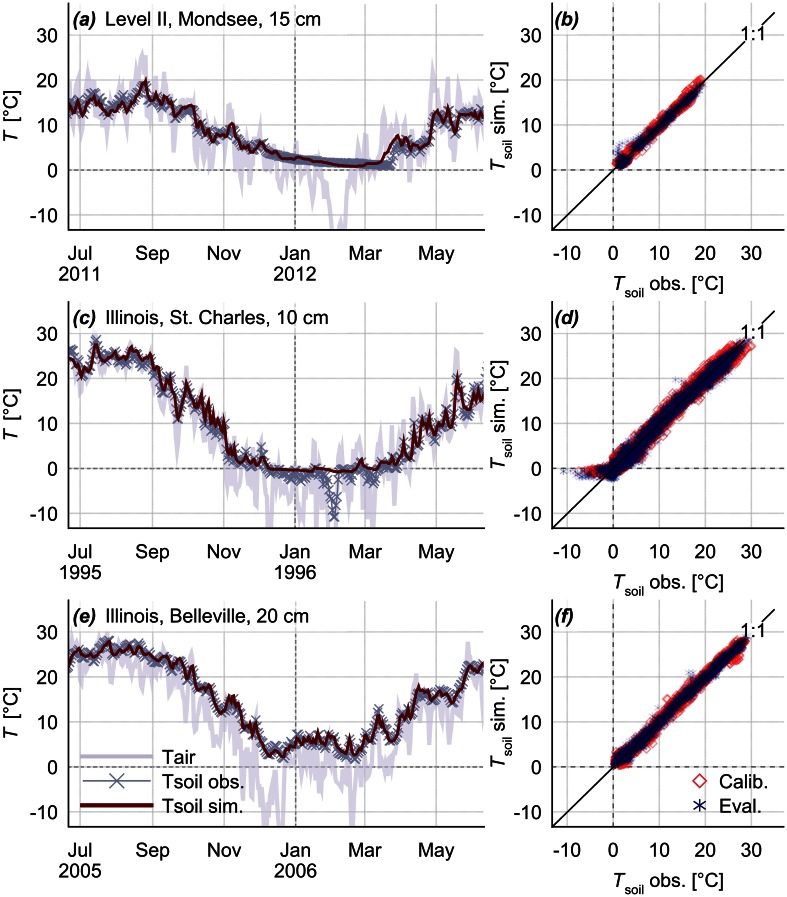
One year section of observed and simulated *T*_soil_ time series plus the corresponding performance scatterplot. Note that the scatterplots cover the whole investigation timeframe! (**a**, **b)** winter snow cover decouples the course of air and soil temperature. The melting of the snowpack in the end of March causes *T*_soil_ to escalate, due to the ceasing insulation plus the abrupt decrease in surface albedo, making the soil susceptible for short wave radiation inputs, which are already considerable in early spring. As the snowpack is not modeled explicitly, the simulator fails to predict the exact time when *T*_soil_ rises in spring (**c**, **d)**. Failure to predict a major soil frost event, due to limitations in the model structure: Temperature fluctuations in early winter indicate the absence of a snow pack. When in midwinter all latent heat is released due to the freezing of soil water, *T*_soil_ suddenly drops. In the formulation the transfer coefficient below the lower threshold temperature (*T*_0_) remains constant. As a consequence, our formulation applies best, to sites where severe soil frost plays only a subordinate role (**e**, **f**)

The model was applied to various sites and depths. In this work, a representative selection of 36 simulation runs is displayed (Table 2). The simulator delivered good estimates of *T*_soil_ on all investigated forest sites. NSE values above 0.979 and RMSE consistently below 1 °C underline the outcome (Table 3), whereat good results were not limited to the topmost soil horizons. Increasing phase shift and the attenuation of the temperature wave with increasing soil depth, were also captured by the simulation (Fig. 3b). Winter *T*_soil_ dynamics are strongly affected by (1) heat transformations at the freeze/thaw transition and (2) the insulating by the snowpack (Beltrami 2001). The presented model does not specifically address these effects, but it is capable, to account for both effects combined. In most cases, the description of the winter soil thermal regime was successful. Figures 3a, d and 4a clearly show the decoupling of ground temperature from air temperature under snow cover. The simulator was able to track this behavior, where in some cases it failed to predict the exact time when soil temperature rises in spring (Fig. 4a): The melting of the snow cover causes a sharp increase in *T*_soil_ due to the ceasing insulating effect, hand in hand with an abrupt decrease in surface albedo, making the forest ground susceptible for short wave radiation inputs, which are already considerable in early spring. Rankinen et al. (2004) solved this problem by incorporating a snow dynamics routine into the calculations, but this would require the embedding of more model parameters and meteorological input data. In consideration of the models practical applicability, this was set aside.

**Table 3:** Performance indices for calibration and evaluation intervals

	Calibration						Evaluation					Timeframe	
	z (cm)	*n*	NSE	RMSE (°C)	MAE (°C)	MBE (°C)	*n*	NSE	RMSE (°C)	MAE (°C)	MBE (°C)	Start	End
Jochberg	15	780	0.991	0.416	0.300	0.003	731	0.989	0.496	0.364	−0.03	03/01/2009	12/31/2013
	30	780	0.991	0.385	0.281	0.003	731	0.990	0.444	0.327	−0.01	03/01/2009	12/31/2013
	60	780	0.992	0.320	0.234	0.003	731	0.991	0.351	0.266	0.013	03/01/2009	12/31/2013
Mondsee	15	761	0.986	0.641	0.513	−0.00	731	0.979	0.806	0.626	0.249	12/01/2009	12/31/2013
	30	761	0.988	0.518	0.409	−0.00	731	0.981	0.678	0.559	0.275	12/01/2009	12/31/2013
	60	761	0.990	0.396	0.301	−0.00	731	0.980	0.570	0.473	0.254	12/01/2009	12/31/2013
Murau	15	1037	0.974	0.604	0.487	−0.00	718	0.986	0.434	0.325	0.017	01/01/2009	12/31/2013
	30	1037	0.974	0.565	0.451	0.002	718	0.987	0.399	0.299	0.045	01/01/2009	12/31/2013
	60	1037	0.970	0.542	0.434	0.001	718	0.989	0.330	0.235	0.037	01/01/2009	12/31/2013
Mürzzuschlag	15	761	0.994	0.381	0.284	0.003	731	0.993	0.441	0.352	0.014	12/01/2009	12/31/2013
	30	761	0.994	0.348	0.261	0.004	731	0.992	0.415	0.314	0.073	12/01/2009	12/31/2013
	60	761	0.994	0.312	0.237	0.010	731	0.994	0.332	0.254	0.079	12/01/2009	12/31/2013
Klausen-Leopoldsdorf	05	1927	0.986	0.625	0.478	−0.00	1770	0.986	0.615	0.482	0.165	11/08/2001	07/01/2013
	10	1927	0.987	0.585	0.448	0.000	1770	0.986	0.581	0.454	0.176	11/08/2001	07/01/2013
	15	1927	0.987	0.550	0.425	−0.00	2126	0.987	0.562	0.438	0.103	11/08/2001	07/01/2013
	30	1927	0.988	0.499	0.386	0.000	2126	0.987	0.527	0.402	0.105	11/08/2001	07/01/2013
	60	1914	0.989	0.418	0.330	−0.00	2126	0.985	0.490	0.375	0.106	11/08/2001	07/01/2013
Unterpullendorf	15	829	0.994	0.492	0.384	−0.00	711	0.992	0.559	0.451	−0.08	09/19/2009	12/31/2013
	30	829	0.995	0.419	0.335	0.004	720	0.994	0.471	0.384	−0.05	09/19/2009	12/31/2013
	60	829	0.995	0.371	0.293	−0.00	720	0.994	0.401	0.317	−0.01	09/19/2009	12/31/2013
HBEF, intensive high	05	489	0.991	0.549	0.402	−0.00	390	0.986	0.704	0.554	0.198	12/01/2010	05/10/2013
HBEF, intensive low	05	493	0.986	0.726	0.560	−0.00	396	0.979	0.943	0.781	−0.68	12/01/2010	05/10/2013
Harvard, Prospect Hill	05	1477	0.989	0.717	0.534	−0.01	1556	0.986	0.811	0.621	0.141	06/01/1991	05/31/2000
Harvard, Barre Woods	05	1290	0.990	0.673	0.513	0.002	1013	0.990	0.664	0.500	−0.18	05/21/2003	04/20/2010
Freeport	10	4568	0.985	1.16	0.864	0.014	4389	0.986	1.07	0.821	−0.06	01/01/1991	07/31/2015
	20	4571	0.989	0.956	0.702	0.001	4389	0.989	0.909	0.703	−0.04	01/01/1991	07/31/2015
St. Charles	10	4524	0.985	1.12	0.870	0.013	4389	0.982	1.19	0.884	0.180	01/01/1991	07/31/2015
	20	4551	0.990	0.875	0.684	0.010	4384	0.987	0.973	0.742	0.160	01/01/1991	07/31/2015
Champaign	10	2190	0.983	1.25	0.984	−0.01	1828	0.982	1.24	0.991	−0.20	01/01/1995	12/31/2005
	20	2190	0.991	0.846	0.686	−0.01	1828	0.989	0.916	0.727	−0.13	01/01/1995	12/31/2005
Belleville	10	2033	0.992	0.784	0.615	0.003	2187	0.991	0.844	0.659	0.098	01/01/2004	07/31/2015
	20	2033	0.991	0.789	0.601	0.002	2193	0.992	0.753	0.584	0.048	01/01/2004	07/31/2015
Brownstown	10	3610	0.985	1.04	0.820	−0.02	3652	0.990	0.845	0.662	0.079	01/01/1991	12/31/2010
	20	3610	0.987	0.950	0.754	0.014	3652	0.990	0.816	0.636	0.070	01/01/1991	12/31/2010
Olney	10	2035	0.989	0.990	0.763	−0.00	2192	0.988	1.02	0.776	−0.02	01/01/2004	07/31/2015
	20	2034	0.992	0.787	0.615	0.009	2192	0.992	0.809	0.621	−0.01	01/01/2004	07/31/2015

Calibration was performed on odd years, evaluation on even ones. A long evaluation timeframe (high *n*) adds significance to the results

A positive MBE indicates that the observed mean soil temperature exceeds the predicted mean soil temperature and vice versa

*NSE* Nash–Sutcliffe efficiency, *RMSE* root mean squared error, *MAE* mean absolute error, *MBE* mean bias error

Compared to forested locations, the biotic site components at the open field meteorological stations are kept intentionally constant. This enabled the successful prediction of *T*_soil_ over a long timeframe. On 2 sites in the northern part of the state (Freeport, St. Charles) we accomplished good results over 24 years of calibration and evaluation. But the best performance (evaluation NSE ≥0.99 over several years) was achieved on comparatively warm locations, located at low elevations, in the south of Illinois (Belleville, Brownstown, Olney). In contrast to forested sites, open field sites, lack the attenuating properties of a dense canopy, or a thick litter layer. Especially for cold, but snow-free winters, these locations were prone to soil frost (Fig. 4c, St. Charles). Temperature fluctuations in early winter indicate the absence of a thick insolating snow pack. When in midwinter all latent heat is released, due to the freezing of soil water, *T*_soil_ suddenly drops. Due to the structure of the model, this behavior could not be tracked: In the presented formulation the transfer coefficient below the lower threshold temperature (*T*_0_) remains constant at a reduced level, suppressing further soil cooling. This model limitation could be tackled by letting the transfer coefficient rise at temperatures below *T*_0_. On the other hand, that would require the segregation of the effects of freeze/thaw processes and snow cover insulation, making the model again more complex and input data demanding.

**Fig. 5:**
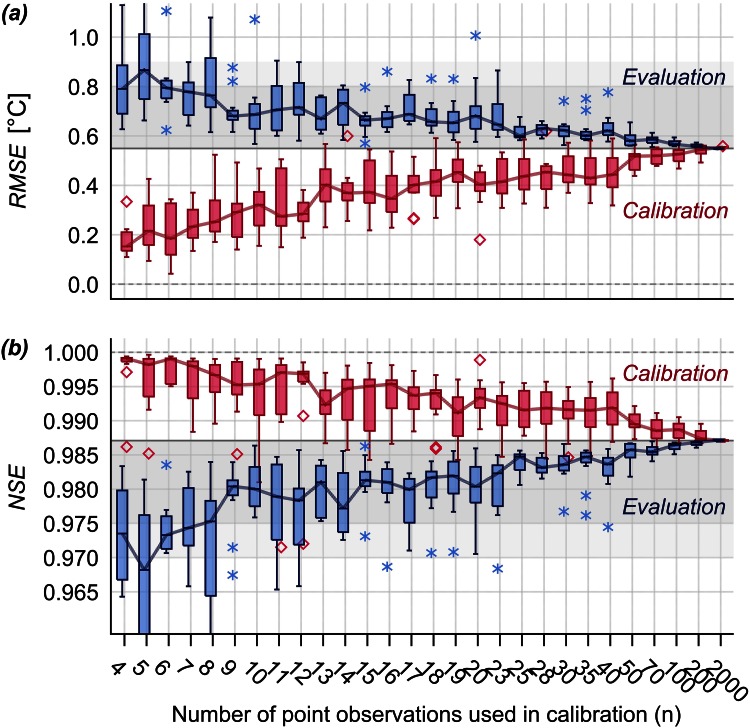
Model optimization result for Klausen-Leopoldsdorf, 15 cm: to determine the amount of point observations, necessary to achieve sound model performance, the investigated time series was divided into *n* intervals of equal size, drawing one random point observation each. These *n* observations were used to optimize the model (simulated annealing). The remaining observations were used to validate model performance. For each *n*, the procedure was repeated 12 times with different random observations, to generate a distributed result. Performance measures shown are (**a**) root mean squared error, and (**b**) Nash–Sutcliffe efficiency. Both indices show high performance (RMSE ≤ 0.9 °C, NSE ≥ 0.97) with *n* ≥ 13. For *n* ≥ 50 there was only little difference in performance, compared to optimization utilizing the full calibration timeframe (*n* = 2000, *horizontal, grey line*)

The examination, to determine the minimum amount of point observations of soil temperature, necessary to yield suitable results, was performed on, an 11-years time series, of air and soil temperature at the Level II plot Klausen-Leopoldsdorf (15 cm depth). The time frame was divided in *n* sectors. Only one observation was selected randomly by sector. All other observations served in the evaluation. Disregarding single outlier runs, good results (NSE >0.97, RMSE < 0.9 °C) were achieved with *n* ≤ 13. Having available 50 or more daily observations, there was only little difference to the result, compared to utilizing ~50 % (*n* = 2000) of the available data in the calibration process (Fig. 5).

Two considerations led to the implementation of decreasing transfer coefficients with decreasing soil temperature: (1) The heat release/consumption at the freeze/thaw transition (Beltrami 2001), and (2) the insulating effect of the winter snow cover. So intentionally, values for *T*_0_ and *T*_1_ were searched around 0 °C. Surprisingly, in most cases the optimization process led to *T*_1_ values much higher, meaning that the attenuation of the transfer coefficient starts already at higher temperatures. The idea behind utilizing different responses for soil warming and cooling, was the assumption, that soil warming in spring is strongly driven by incoming solar radiation, which is accelerating the temperature rise.

**Fig. 6:**
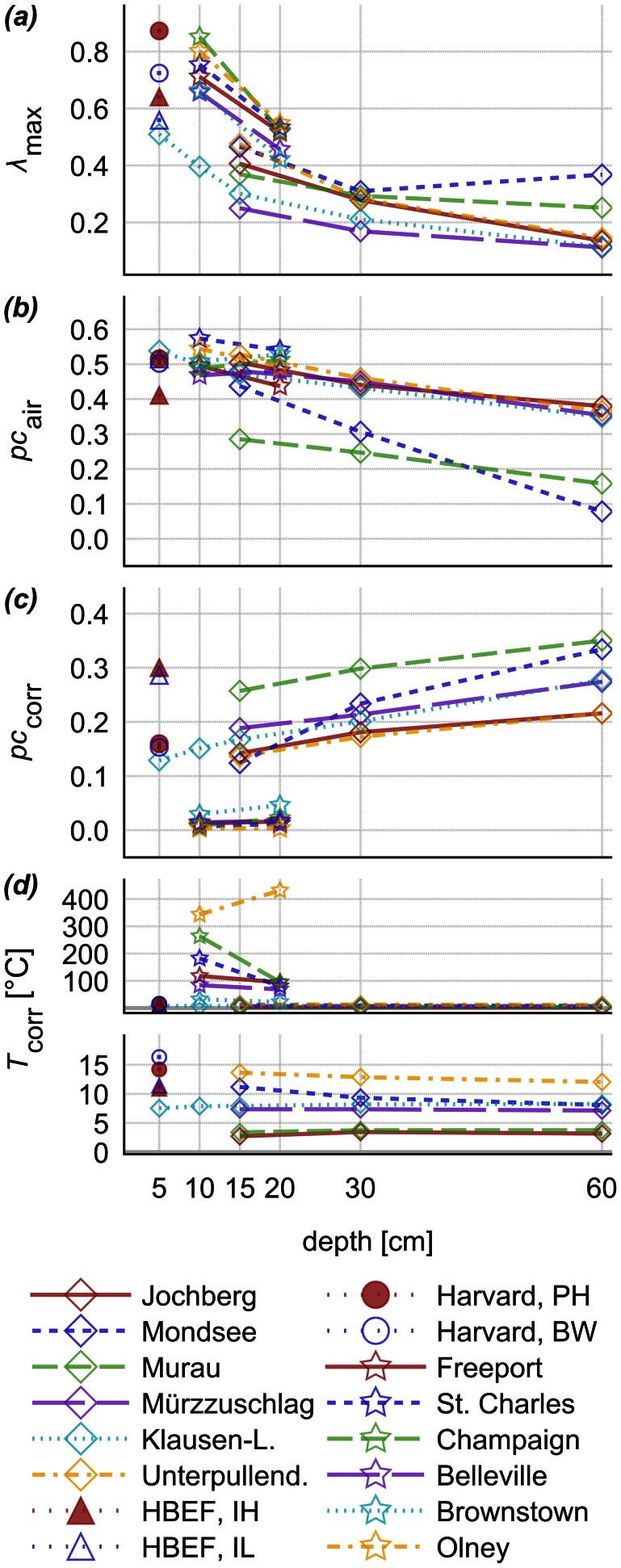
Four selected parameters and their change with increasing soil depth. **a** Transfer coefficient values showed a clear decreasing trend with increasing soil temp. **b** Also the fraction of the air temperature in the calculation of the environmental temperature showed, almost linear, decrement. **d** Where the correction temperature on forested sites was in a close range to the annual mean air temperature, the open field locations (*star symbol*) revealed much higher values. On the other hand, the relative weighting (**c**) of these temperatures was much smaller on non-forested sites. It is assumed, that on these locations, both parameters combined compensate for direct shortwave radiation inputs

As this model is primarily of an empirical nature, used parameters lack a specific meaning, in a strict physical sense. Nevertheless, it was noted that parameter values were strongly affected by certain site characteristics: *λ*_max_ values clearly decreased with increasing soil depth (Fig. 6a). Meaning, the time demand, to compensate a fraction of the temperature difference between soil layer and air, rose with increasing soil depth. Also the relative partition of the correction temperature (*pc*_corr_), in the calculation of the environmental temperature, increased in deeper soil layers (Fig. 6c). In contrast, the direct influence of air temperature (*pc*_air_) showed a decrease downwards.

Investigated open field sites differed strongly from forest sites, in parameter values of the correction temperature (*T*_corr_). Where on forest locations *T*_corr_ resided closely to the stands annual mean air temperature, open field sites revealed *T*_corr_ values, around and above 100 °C (Fig. 6d). On the other hand, their relative weight (*pc*_corr_) in the calculation of the environmental temperature, is much lower than on forested sites. It is assumed, that in these cases, they correct for direct radiation energy inputs, which are obviously much higher without the presence of a shielding canopy. The reason that, even under such conditions, the simulator (which does not particularly address radiative heat flux) delivers good estimates of *T*_soil_, might be found in the strong correlation between energy balance components, and the air temperature itself (Hock 2003).

## Conclusion

The primary intention of this work was the provision of a tool, which enables the transformation of fragmentary records of forest soil temperature, into a complete time series of *T*_soil_, using average daily air temperature as only input. In this specific case, the created time series is laying the base for the modeling of temperature dependent, biogeochemical soil processes.

To test the resilience of this model, it was applied to various locations and depths, covering a broad amplitude of site characteristics. The simulator delivered accurate predictions of the temperature of the topsoil, as well as of deeper layers. The high performance was not limited to the warm season. The combination of the insulating effect of the snow cover plus the effect of heat transformations at the freeze/thaw transition, on soil thermal regimes were captured sufficiently. The formulation was applied to forested, as well as open to field locations, where in the open field it failed to reproduce some major soil frost events. Bearing this limitation in mind, this simulator seems to be well applicable to other land use types.

The model parameters lack a specific meaning in a strict physical sense. Therefore, currently the parameterization requires at least a modest amount of *T*_soil_ observations, to yield sufficient results. A challenging impulse for future work, would be the attempt to derive model parameters, directly from more easily obtainable site characteristics. This also would enable the capability of the simulator to deal with a changing soil thermal regime, during stand development.
